# Understanding the ethical and legal considerations of Digital Pathology

**DOI:** 10.1002/cjp2.251

**Published:** 2021-11-18

**Authors:** Cheryl Coulter, Francis McKay, Nina Hallowell, Lisa Browning, Richard Colling, Philip Macklin, Tom Sorell, Muhammad Aslam, Gareth Bryson, Darren Treanor, Clare Verrill

**Affiliations:** ^1^ Department of Cellular Pathology Oxford University Hospitals NHS Foundation Trust, John Radcliffe Hospital Oxford UK; ^2^ Nuffield Division of Clinical Laboratory Sciences University of Oxford, John Radcliffe Hospital Oxford UK; ^3^ The Wellcome Centre for Ethics and Humanities and the Ethox Centre, Nuffield Department of Population Health University of Oxford Oxford UK; ^4^ NIHR Oxford Biomedical Research Centre Oxford University Hospitals NHS Foundation Trust, John Radcliffe Hospital Oxford UK; ^5^ Nuffield Department of Surgical Sciences University of Oxford, John Radcliffe Hospital Oxford UK; ^6^ Department of Politics and International Studies University of Warwick Coventry UK; ^7^ Department of Histopathology Glangwilli Hospital, Hywel Dda University Health Board Carmarthen Wales, UK; ^8^ Department of Pathology Queen Elizabeth University Hospital, NHS Greater Glasgow and Clyde Glasgow Scotland, UK; ^9^ Department of Pathology Leeds Teaching Hospitals NHS Trust Leeds UK

**Keywords:** Digital Pathology, governance, ethics, legal, training, histopathology

## Abstract

Digital Pathology (DP) is a platform which has the potential to develop a truly integrated and global pathology community. The generation of DP data at scale creates novel challenges for the histopathology community in managing, processing, and governing the use of these data. The current understanding of, and confidence in, the legal and ethical aspects of DP by pathologists is unknown. We developed an electronic survey (e‐survey), comprising 22 questions, with input from the Royal College of Pathologists (RCPath) Digital Pathology Working Group. The e‐survey was circulated via e‐mail and social media (Twitter) through the RCPath Digital Pathology Working Group network, RCPath Trainee Committee network, the Pathology image data Lake for Analytics, Knowledge and Education (PathLAKE) digital pathology consortium, National Pathology Imaging Co‐operative (NPIC), local contacts, and to the membership of both The Pathological Society of Great Britain and Ireland and the British Division of the International Academy of Pathology (BDIAP). Between 14 July 2020 and 6 September 2020, we collected 198 responses representing a cross section of histopathologists, including individuals with experience of DP research. We ascertained that, in the UK, DP is being used for diagnosis, research, and teaching, and that the platform is enabling data sharing. Our survey demonstrated that there is often a lack of confidence and understanding of the key issues of consent, legislation, and ethical guidelines. Of 198 respondents, 82 (41%) did not know when the use of digital scanned slide images would fall under the relevant legislation and 93 (47%) were ‘Not confident at all’ in their interpretation of consent for scanned slide images in research. With increasing uptake of DP, a working knowledge of these areas is essential but histopathologists often express a lack of confidence in these topics. The need for specific training in these areas is highlighted by the findings of this study.

## Introduction

The past 20 years have witnessed the growing digitalisation of histopathology services as a result of developments in whole slide image (WSI) scanning, storage, and analysis [1]. The implementation of Digital Pathology (DP) has been relatively slow in the United Kingdom (UK), although rapid changes are on the horizon [2, 3]. Currently, there are 41 pathology units in the UK, with approximately 1256 consultants. A UK 2018 survey found that 60% of pathology units have access to a DP scanner [3]. Following recent investment in DP infrastructure, five Artificial Intelligence (AI) Centres of Excellence have been established in the UK, aiming to develop the country's DP and imaging expertise. These are: the Industrial Centre for AI Research in Digital Diagnostics (I‐CAIRD) based in Glasgow, the London Medical Imaging and Artificial Intelligence Centre for Value‐Based Healthcare, the National Consortium of Intelligent Medical Imaging (NCIMI) in Oxford, the National Pathology Imaging Co‐operative (NPIC) based in Leeds, and the Pathology image data Lake for Analytics, Knowledge and Education (PathLAKE) based in Coventry.

The adoption of DP is part of the strategy set out by the *NHS Long Term Plan* for digitally enabled care and the UK Government's *Industrial Life Sciences Strategy* [4, 5]. Stakeholders relevant to the digitalisation of pathology in the UK include The National Data Guardian, The Information Commissioner's Office (ICO), Alan Turing Institute, Health Data Research UK, Care Quality Commission, Public Health England, NHS Digital, and NHSX [6, 7, 8, 9, 10, 11, 12, 13]. Internationally, the World Health Organisation (WHO), in partnership with the International Telecommunication Union (ITU), has established a Focus Group on Artificial Intelligence for Health (FG‐AI4H) with the aim of identifying opportunities for standardisation and applications of AI to health issues on a global scale [14].

DP involves the creation of digital images by using a scanning device to provide high‐resolution images that can be viewed on a platform. This provides a stage platform for developing and utilising AI algorithms, for recognising subtle patterns in tissue to assist diagnosis or derive novel insights into disease [15, 16, 17, 18]. Generation of data from DP creates challenges for the histopathology community with regard to data management, processing, and security. The UK Government has stated that protecting patient data is a legal requirement of paramount importance [6, 19]. This is echoed globally, with guidelines, policies, and legislations aiming to ensure appropriate application of DP (Table 1).

**Table 1:** DP – relevant guidelines, position papers, regulations, and legislation relating to the management/use of data generated through WSI (amended from García‐Rojo [20] and Chong *et al* [21], non‐exhaustive list).

Country/region	Guideline/legislation	Comments
UK	2018: Royal College of Pathologists – Best practice recommendations for implementing DP	Provides ‘an overview of the technology involved in DP and of the currently available evidence on its diagnostic use, together with practical advice for pathologists on implementing DP’ [22]
	2018: UK Government – The DPA	Stipulates how personal information is used by organisations, businesses, or the government. It is the UK's implementation of the GDPR [23]
	2018: UK Government – NHS Data Opt‐Out	Introduced to enable patients to opt out from the use of their data for research or planning purposes in line with the recommendations of the National Data Guardian [24]
	2018: UK Government – Code of Conduct for Data Driven Health and Care Technology	A guide to good practice for the use of digital technology in health and care. The guide provides a set of principles that state what is expected from suppliers and users of data‐driven technologies [19]
	2019: UK Government – NHSX Artificial Intelligence How to Get it Right	Provides an overview of the current state of play of data‐driven technologies within the health and care system in the UK [6]
	Ongoing: Office for National Statistics (ONS): Principles for Data Initiatives	ONS is the UK's largest independent producer of official statistics, responsible for collecting and publishing statistics related to population and society. The *Principles for Data Initiatives* is a section of the ONS Data Strategy, which states their fundamental principles and standards to promote public trust in their data handling [25]
	Ongoing: Common Law Duty of Confidentiality	Common law (case law) is law that has developed through the courts making decisions in cases on legal points and creating binding precedents in contrast to statutory law which is determined by acts of parliament. It is the legal obligation for confidentiality; when personal information is shared in confidence, it must not be disclosed without some form of legal authority or justification [26]
EU	2021: EU: Medical Devices Regulation	Regulation stating that software will be considered a medical device if it forms part, or is an accessory, of a medical device or where it constitutes standalone software, has a medical purpose, and the processing of the data goes beyond mere storage, archiving, communication, or simple search [27]
	2018: EU: GDPR	Regulation drafted and passed by the EU for the processing of personal information, either within the EU or information related to people in the EU [28]
	2016: EU – US Privacy Shield	It was a framework for regulating transatlantic exchanges of personal data for commercial purposes between the EU and US. In 2020, a court issued that the framework no longer provided adequate safeguards so is now defunct [29]
The United States of America	2021: Healthcare and Public Health Sector Coordinating Council (HSCC) Position Paper	The HSCC Joint Cybersecurity Working Group is a standing working group of the HSCC composed of more than 300 industry and government organisations working together to develop strategies to address emerging and ongoing cybersecurity challenges to the health sector. They do state that the federal and state regulations have not kept in step with the rapid and widespread adoption of telehealth technologies across the country. Currently, there is no single federal agency with authority to establish and enforce privacy and security requirements for the entire telehealth ecosystem [30]
	2021: College of American Pathologists – Validating Whole Slide Imaging Systems for Diagnostic purposes in Pathology, Guidelines Update	Guidelines stating if WSI is used for diagnostic or other related clinical purposes, procedures must be in place that ensure sites using WSI provide reasonable and expected confidentiality and data security, in both data storage and data transmission [31]
	2020: US Food and Drug administration (FDA) – Enforcement Policy for remote DP devices during the Coronavirus Disease 2019 Public Health Emergency	Previously, FDA‐approved WSI devices were not cleared for home use or categorised as waived by FDA, so limited to use in clinical laboratories and their healthcare settings. In March 2020, the Centers for Medicare & Medicaid Services (CMS) issued a memorandum, describing its exercise of enforcement discretion to ensure pathologists may review pathology slides and images remotely [32]
	2020: American Telemedicine Association (ATA). Policy Principles	Policies highlighting the importance of protection of patient privacy and cybersecurity risks along with the importance of ensuring safe transfer across state lines. Not specific for DP [33]
	2019 (initially authorised 2017): US FDA	WSI device authorised for marketing in the US with a second system cleared for use in 2019 [34]
	2018: ATA Clinical Guidelines for Telepathology	Guidelines state that all data transmission used in telepathology should be secured through the use of encryption that meets recognised standards. The ATA also recommends that protected health information and other confidential data only be backed up to or stored on secure data storage locations. Cloud services unable to achieve compliance should not be used for personal health information or confidential data [35]
	2015: United States Government: Cybersecurity Information Sharing Act	Established a mechanism for cybersecurity information sharing among private sector and federal government entities – provides a set of cybersecurity best practices that should be used in the protection of telehealth and telemedicine systems and services [36]
	1996: US Department of Health and Human Services. Health Insurance Portability and Accountability Act (HIPAA)	The act mandates data security and privacy controls to keep medical information safe. The Department of Health and Human Services (HHS) publishes the HIPAA privacy rule, the HIPAA security rule, and the HIPAA breach notification rule [37]
Canada	2019: Office of the Privacy Commissioner of Canada – The Personal Information Protection and Electronic Documents Act (PIPEDA)	The PIPEDA applies to private sector organisations across Canada that collect, use, or disclose personal information in the course of a commercial activity. Personal information relating to hospitals can also be covered by provincial laws [38]
	2014: Canadian Association of Pathologists – Guidelines for establishing a telepathology service for anatomical pathology using WSI	The objective is to provide Canadian pathologists with baseline information on how to implement and use relevant platforms. Guidelines cover privacy and security, document, and archiving and liability [39]
	2005: Canadian Association of Pathologists – Code of ethics for storage and transmission of electronic laboratory data	A voluntary code based on the work of the Guidelines Governing the Protection of Privacy and Transborder Flows of Personal Data, created by the international Organization for Economic Cooperation and Development (OECD) [40]
Germany	2018: Professional Association of German Pathologists – Digital Pathology in Diagnostics – reporting on digital images	Purpose of the guidelines is to direct the framework on how to implement virtual microscopy in routine diagnosis in Germany and includes the topic of data security [41, 42]
Australasia	2015: The Royal College of Pathologists of Australasia (RCPA) – Guidelines for Digital Microscopy in Anatomical Pathology and Cytology	Guidelines include a module on ‘Privacy, Confidentiality, and Security’, which states that system must comply with national and state privacy regulations and is determined by the Privacy Act 1988 that regulates how personal information is handled and includes 13 Australian Privacy Principles [43]
Spain	2021: The Spanish Society of Pathology – White Paper 2021 of the Pathological Anatomy in Spain	Guidelines include acknowledgement that ‘The storage system of digital preparations must be based on open solutions and in international standards…. which will facilitate compliance with the Regulation GDPR’ [44]
South Korea	2020: Korean Society of Pathologists (KSP) – Recommendations for pathological practice using DP	The guidelines include ‘strict technical measures must be in place to ensure information security and protect personal information regardless of the type of terminal being used. Therefore, measures are needed to ensure that transmitted data are not easily released outside the network and that transmitted metadata do not contain personal information to minimise the risk to personal data even if a data leak was to occur’ [21]

Literature discussing the potential benefits of DP/AI rarely mention the ethical and legal considerations of access to, and processing of, patient data [45, 46, 47, 48, 49, 50, 51]. Advice/guidance on medical ethics and data governance exists, although the depth to which these topics are explored varies significantly (Table 1). This is complicated by WSI data often being generated primarily for diagnostic purposes but having other uses, i.e. teaching and research. This complex situation is compounded when collaborating with industry partners, and by political uncertainty such as the UK's recent withdrawal from the European Union (EU) and working across jurisdictions. It is unclear whether present frameworks and guidelines are useful to histopathologists or if there is a need for additional pathology‐focused guidelines [6, 28, 52, 53, 54, 55].

In this paper, we present the results of an electronic survey (e‐survey) aiming to evaluate UK histopathologists' current levels of understanding of, and confidence with, the legal and ethical aspects of DP and their perceived training needs. We evaluate whether the rise of DP has created this need with the move from tissue‐based work (where there is experience with legislation) to image/data‐centred practice with different considerations; although targeted specifically at DP rather than AI, we also discuss relevant overlapping issues.

## Materials and methods

We developed an e‐survey comprising 22 questions using the online platform ‘SurveyMonkey’ (www.surveymonkey.co.uk). The questionnaire (supplementary material, Appendix S1) was developed with input from the Royal College of Pathologists (RCPath) Digital Pathology Working Group.

Information provided to gain consent included: study purpose, approximate completion time, confirmation of anonymity, and details of principal investigators. ‘SurveyMonkey’ collected the data; their privacy policy was present on their website at the time of completion. Consent was implicit by completion of the survey. No ethics approval was required.

Initially, a pilot e‐survey was developed to assess the usability and technical functionality of the e‐survey and tested by a pre‐selected cohort (three consultant histopathologists and one specialty trainee) as a closed survey. The e‐survey was structured with single sequential questions and question formats included: forced‐choice, Likert scales, yes/no options, and open‐ended/free text questions. The topics were: Demographics, Background, Training, Guidelines and Legislation, Consent, and Data sharing. Participants could review and amend answers prior to submission. Following the pilot e‐survey, questions were rephrased to avoid testing specific knowledge and to gauge general understanding.

Following satisfactory completion of the pilot e‐survey, the open e‐survey web link was circulated via social media (Twitter) and e‐mail through the RCPath Digital Pathology Working Group network, RCPath Trainee Committee network, the PathLAKE digital pathology consortium, National Pathology Imaging Co‐operative (NPIC), and to membership of The Pathological Society of Great Britain and Northern Ireland and the British Division of the International Academy of Pathology (BDIAP). The survey was voluntary with no incentives offered. The survey link remained active between 14 July 2020 and 6 September 2020 and was closed only after several reminders had been sent out; only a few additional responses were gathered. The responses were automatically collected by the ‘SurveyMonkey’ platform which has mechanisms to prevent duplication by individuals.

Survey responses were analysed using descriptive statistics. Inductive analysis of free text answers (supplementary material, Appendix S1 – Questions 20 and 22) was performed using open coding with a final coding frame developed specifically for this study. Both sections were coded together and went through two independent rounds of open coding to determine the relevant themes. The codes were then cross referenced to determine the following four main meta‐categories: Governing Data, Validating Data, Ownership and Third‐Party Access, and Inclusivity and Transparency. Ambiguous and uncategorised comments were not reported. This manuscript has been prepared in accordance with the *CHERRIES* guidelines [56].

## Results

### Response

In total, we received 198 responses including 194 histopathologists. Three advanced biomedical scientists and one clinical scientist also completed the survey, although it was stated that the survey was targeted at histopathologists specifically. As these responses represented only 2% of the overall responses, they were included in the analysis and we postulate that they represent scientists who use DP in their role. There was an overall completion rate of 79% with minor variations in response rates to individual questions having been indicated.

Consultants and specialty doctors with >20 years of clinical experience were the most common responders (35%, 70/198). Most respondents were based in England, working in the NHS tertiary referral centres in NHS posts with no funded academic time (Table 2). The most common experience with DP was ‘External Quality Assurance (EQA)’ (82%, 160/196), followed by ‘Teaching or training’ (72%, 142/196). The most common experience with consultants was ‘EQA’ (93%, 136/146), whereas for trainees it was ‘Teaching or training’ (94%, 44/47) (Table 2). Forty‐three percent (85/198) of respondents had been involved in research using digital scanned slides; a further 6% (12/198) were planning to do so in the future. Two percent (4/196) had no experience in any of the stated areas of DP.

**Table 2:** Summary of respondents – grade, region, current post, and current centre.

Question	Responses
Question 1. Current level of experience	
Consultant histopathologist with >20 years' experience*	35% (70/198)
Consultant histopathologist with 15–20 years' experience*	17% (33/198)
Consultant histopathologist with 10–15 years' experience*	13% (25/198)
Consultant histopathologist with 5–10 years' experience*	10% (19/198)
Trainee histopathologist	24% (47/198)
Non‐histopathologist	3× advanced biomedical scientists and 1× clinical scientist
Question 2. Region	
England	81% (160/198)
Scotland	12% (24/198)
Wales	4% (8/198)
Northern Ireland	3% (6/198)
Question 3. Current post	
NHS post, no funded academic time	76% (149/195)
NHS post, including some funded academic time	11% (22/195)
Academic post including some funded NHS time	11% (21/195)
Academic post, no funded NHS time	2% (3/195)
Question 4. Current centre	
NHS – district general hospital	30% (59/198)
NHS – tertiary referral centre	35% (70/198)
NHS centre and university academic department	31% (61/198)
University academic department	4% (7/198)
Private laboratory	1% (1/198)
Question 5. Experience with DP	
Primary diagnosis	
Consultant histopathologist with 5 to >20 years' experience*	36% (53/146)
Trainee histopathologist	23% (11/47)
Non‐histopathologist	0% (0/3)
Second opinion	
Consultant histopathologist with 5 to >20 years' experience*	24% (35/146)
Trainee histopathologist	9% (4/47)
Non‐histopathologist	0% (0/3)
Multidisciplinary team meeting	
Consultant histopathologist with 5 to >20 years' experience*	34% (50/146)
Trainee histopathologist	13% (6/47)
Non‐histopathologist	67% (2/3)
Research or clinical trials	
Consultant histopathologist with 5 to >20 years' experience*	38% (55/146)
Trainee histopathologist	26% (12/47)
Non‐histopathologist	67% (2/3)
Teaching	
Consultant histopathologist with 5 to >20 years' experience*	65% (95/146)
Trainee histopathologist	94% (44/47)
Non‐histopathologist	67% (2/3)
EQA	
Consultant histopathologist with 5 to >20 years' experience*	93% (136/146)
Trainee histopathologist	49% (23/47)
Non‐histopathologist	33% (1/3)
No experience of specific DP activities	
Consultant histopathologist with 5 to >20 years' experience*	1% (2/146)
Trainee histopathologist	4% (2/47)
Non‐histopathologist	0% (0/3)
Question 12. Involvement in research	
Overall respondents	
Yes	43% (85/198)
No	51% (101/198)
Planning to undertake research	6% (12/198)
Grade of those involved in research	
Consultant histopathologist with >20 years' experience*	35% (30/85)
Consultant histopathologist with 15–20 years' experience*	19% (16/85)
Consultant histopathologist with 10–15 years' experience*	13% (11/85)
Consultant histopathologist with 5–10 years' experience*	8% (7/85)
Trainee histopathologist	22% (19/85)
Non‐histopathologist	2% (2/85)

* Or specialty doctor, including training.

### Training

Eighty‐eight percent of respondents (168/190) working in an NHS centre (190/198) reported having been offered NHS mandatory training in information governance, 4% (8/190) had not, and the remaining 7% (14/190) were ‘Not sure’. Nearly a quarter of all respondents (24%, 48/198) had undertaken additional training in information governance. Respondents reported attending a variety of additional online and face‐to‐face courses over the years (Table 2).

When asked about their need/desire for training, 39% (27/70) of ‘consultants and specialty doctors with over 20 years' experience’ wanted additional training, whereas approximately half of those with 5–20 years' experience (51%, 39/77) and trainees (51%, 24/47) wanted additional training. Those wanting further training called for this to be specific to DP and to be delivered in an online Continuing Professional Development (CPD)‐accredited format. Summary of current available training, format, and content proposed by respondents is presented in Table 3.

**Table 3:** Summary of respondents' comments regarding additional training.

Question 7. Current additional training	‘Personal Information Commissioner Officer registration’ ‘Education for information commissioner registration and duties under the Data Protection Act 2018’ ‘e‐learning as General Medical Council (GMC) associate’ ‘The Oxford University Information Governance online modules’ ‘Part of master's degree’ ‘General Data Protection Regulation (GDPR) sessions’ ‘Information governance training as part of Good Clinical Practice (GCP) course’ ‘Medical Research Council (MRC) module’ ‘One day generic General Data Protection Regulation (GDPR) course’
Question 8. Proposed additional training; format and content (41 respondents provided additional comments)	*Format* Online modules + webinar Standalone webinar PowerPoint e‐learning modules Continuing Professional Development (CPD) accreditation
	*Content* Healthcare‐specific GDPR Specific to DP Theory of the legal and ethical considerations of DP Application of legislation Examples including case reports/exemplars Use of scanned slide image sharing Anonymising cases – when and how to Dos and don'ts of DP Templates/guidelines Risks and responsibilities Relevance to development of AI tools Implications of reporting patient specimens off site Use of images in publication and online education

### Confidence in applying guidelines/legislation

Table 4 outlines respondents' confidence ratings concerning their understanding of policy/legislations and ethical guidelines. The *Data Protection Act 2018* (*DPA 2018*) had the highest confidence ratings [23]. At the other end of the spectrum, respondents were least confident in their knowledge of the (now defunct) *EU – US Privacy Shield*, followed by the *NHS National Data Opt‐out* [24, 29]. Regarding ethical guidance, all three groups reported predominantly ‘Not at all confident’ in their understanding of all three guidelines. There seemed to be higher confidence levels for legislation (General Data Protection Regulation [GDPR] and DPA 2018) compared to ethical guidance [6]. Comparing responses of those who had or had not received additional training revealed confidence ratings were higher across respondents who had undergone additional training.

**Table 4:** Response rates on confidence rating for legal (Question 9) and ethical guidance (Question 10); overall, comparing those with additional training to those without and comparing those involved in research to those not involved in research (excludes those reporting that they are not currently involved in research but are planning to in the future).

	Not confident at all (%)	Slightly confident (%)	Somewhat confident (%)	Fairly confident (%)	Completely confident (%)	I am not aware of the policy/legislation (%)	Total number of responses	
Legal policy/legislation								
GDPR	16	23	27	24	8	1	196	Overall
	13	4	28	30	20	4	46	Additional training
	17	29	27	22	5	0	150	Without additional training
	8	18	27	34	12	1	83	Involved in research
	24	25	27	18	6	1	101	Not involved in research
DPA 2018	19	22	22	25	10	2	197	Overall
	15	13	21	28	19	4	47	Additional training
	21	25	27	24	7	1	150	Without additional training
	14	17	20	36	12	1	84	Involved in research
	23	28	22	17	9	2	101	Not involved in research
Health and Social Act 2018	38	20	15	12	5	9	195	Overall
	30	17	20	15	13	4	46	Additional training
	41	21	13	11	3	11	149	Without additional training
	33	16	17	17	6	12	83	Involved in research
	44	22	13	8	5	8	100	Not involved in research
EU – US Privacy Shield	57	8	8	5	2	20	197	Overall
	43	9	21	9	2	17	47	Additional training
	62	8	4	4	1	21	150	Without additional training
	50	8	11	8	0	23	84	Involved in research
	63	7	7	3	3	18	101	Not involved in research
NHS Data Opt‐Out	46	20	9	10	3	12	196	Overall
	30	26	15	11	9	9	46	Additional training
	51	18	7	10	1	13	150	Without additional training
	37	24	7	13	4	14	83	Involved in research
	53	18	10	7	3	10	101	Not involved in research
Common Law Duty of Confidentiality	29	17	15	27	7	7	198	Overall
	21	17	13	27	13	10	48	Additional training
	31	17	15	27	5	5	150	Without additional training
	20	16	16	32	8	7	85	Involved in research
	35	17	14	23	6	6	101	Not involved in research
Ethical guidance								
Code of Conduct for Data Driven Health and Care Technology 2018	57	8	7	4	2	23	198	Overall
	42	8	10	8	4	27	48	Additional training
	61	7	6	2	1	22	150	Without additional training
	48	9	9	4	4	26	85	Involved in research
	64	7	5	4	1	20	101	Not involved in research
NHSX: AI: How to Get it Right. 2019	59	6	5	4	1	26	198	Overall
	38	10	8	8	4	31	48	Additional training
	66	4	3	3	0	24	150	Without additional training
	51	7	7	7	2	26	85	Involved in research
	67	4	3	2	0	25	101	Not involved in research
Data Ethics Framework by Department for Digital Culture, Media and Sport	62	7	3	4	0	25	198	Overall
	48	8	4	8	0	31	48	Additional training
	66	6	3	2	0	23	150	Without additional training
	55	8	5	6	0	26	85	Involved in research
	68	5	2	2	0	24	101	Not involved in research

### Understanding of the UK legislation and application for REC approval

Forty‐one percent (82/198) of respondents selected ‘I do not know’ in connection with circumstances where the use of digital scanned slide images would fall under relevant legislation (i.e. GDPR and DPA 2018). Approximately one‐third of research active respondents (31%, 26/85) stated that they did not know when a particular use is covered by legislation versus 50% (50/101) of non‐researchers. The responses are summarised in Table 5.

**Table 5:** Response rates (Question 13) when asked, ‘when would the use of digital scanned slide images fall under the relevant UK data protection legislation (General Data Protection Regulation and Data Protection Act)? (Please tick all that apply)’.

	Response rate
Overall	
‘It always does’	22% (44/198)
‘If there is a patient name on the slide label’	35% (69/198)
‘If there is a histology (accession) number on the slide’	25% (50/198)
‘The slide is fully anonymised (link to the case permanently broken)’	6% (11/198)
‘The slide is pseudonymised (personal identifiers are removed, but a link or key to identify the case remains)’	23% (46/198)
‘I do not know’	41% (82/198)
+/− Research	
Have undertaken Digital Pathology research and ‘It always does’	26% (22/85)
Have NOT undertaken Digital Pathology research and ‘It always does’	20% (20/101)
Have undertaken Digital Pathology research and ‘I do not know’	31% (26/85)
Have NOT undertaken Digital Pathology research and ‘I do not know’	50% (50/101)
Involved in research	
‘If there is a patient name on the slide label’	39% (33/85)
‘If there is a histology (accession) number on the slide’	29% (25/85)
‘The slide is fully anonymised (link to the case permanently broken)’	5% (4/85)
‘The slide is pseudonymised (personal identifiers are removed, but a link or key to identify the case remains)’	31% (26/85)
Not involved in research	
‘If there is a patient name on the slide label’	31% (31/101)
‘If there is a histology (accession) number on the slide’	22% (22/101)
‘The slide is fully anonymised (link to the case permanently broken)’	7% (7/101)
‘The slide is pseudonymised (personal identifiers are removed, but a link or key to identify the case remains)’	17% (17/101)

**Figure 1:**
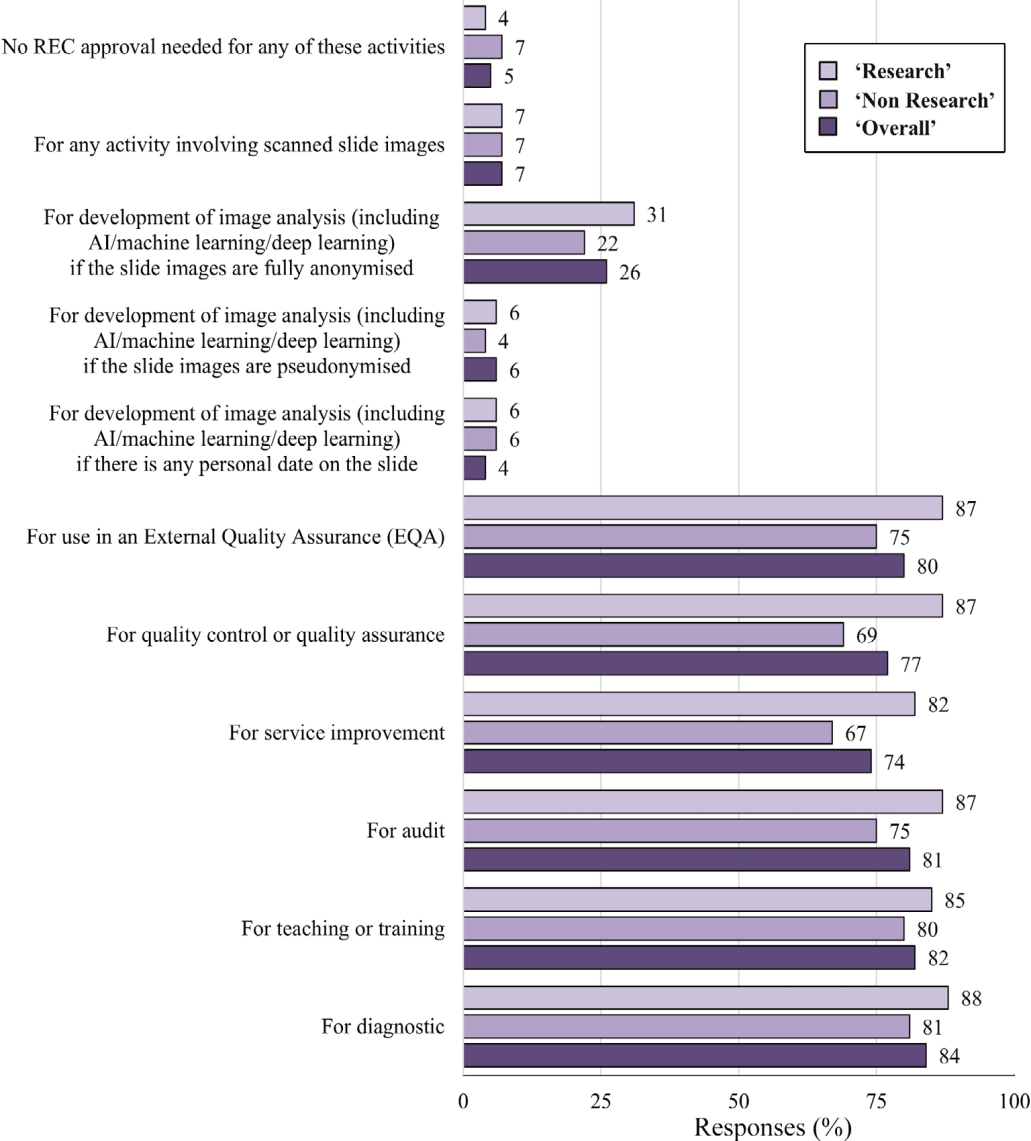
The percentage response rates to Question 14: ‘Which of the following activities, using digital scanned slides/platform, would you NOT require Research Ethics Committee (REC) approval for? (Please tick all that apply)’.

With regard to the Research Ethics Committee (REC), we asked under which circumstances would one *not* require REC approval for the use of digital images. The majority of respondents answered ‘Diagnostic reporting’ (84%), ‘Teaching or training’ (82%), ‘Audit’ (81%), and ‘In an EQA’ (80%). Five percent responded that ‘No REC approval’ was required for any of the proposed activities (Figure 1).

### Consent

Forty percent (79/197) of all respondents stated ‘I do not know’ when asked if there was a statement on the consent procedure/investigation form in their NHS Trust for the use of data and/or tissue in research. Research active respondents were more familiar with this statement as would be expected as it is of less relevance to non‐research active pathologists. Only 25% (21/85) of those undertaking research responded with ‘I do not know’ compared to 51% (52/101) of those not undertaking research. Interestingly, 6% (12/197) of overall respondents reported that there was no such statement.

**Figure 2:**
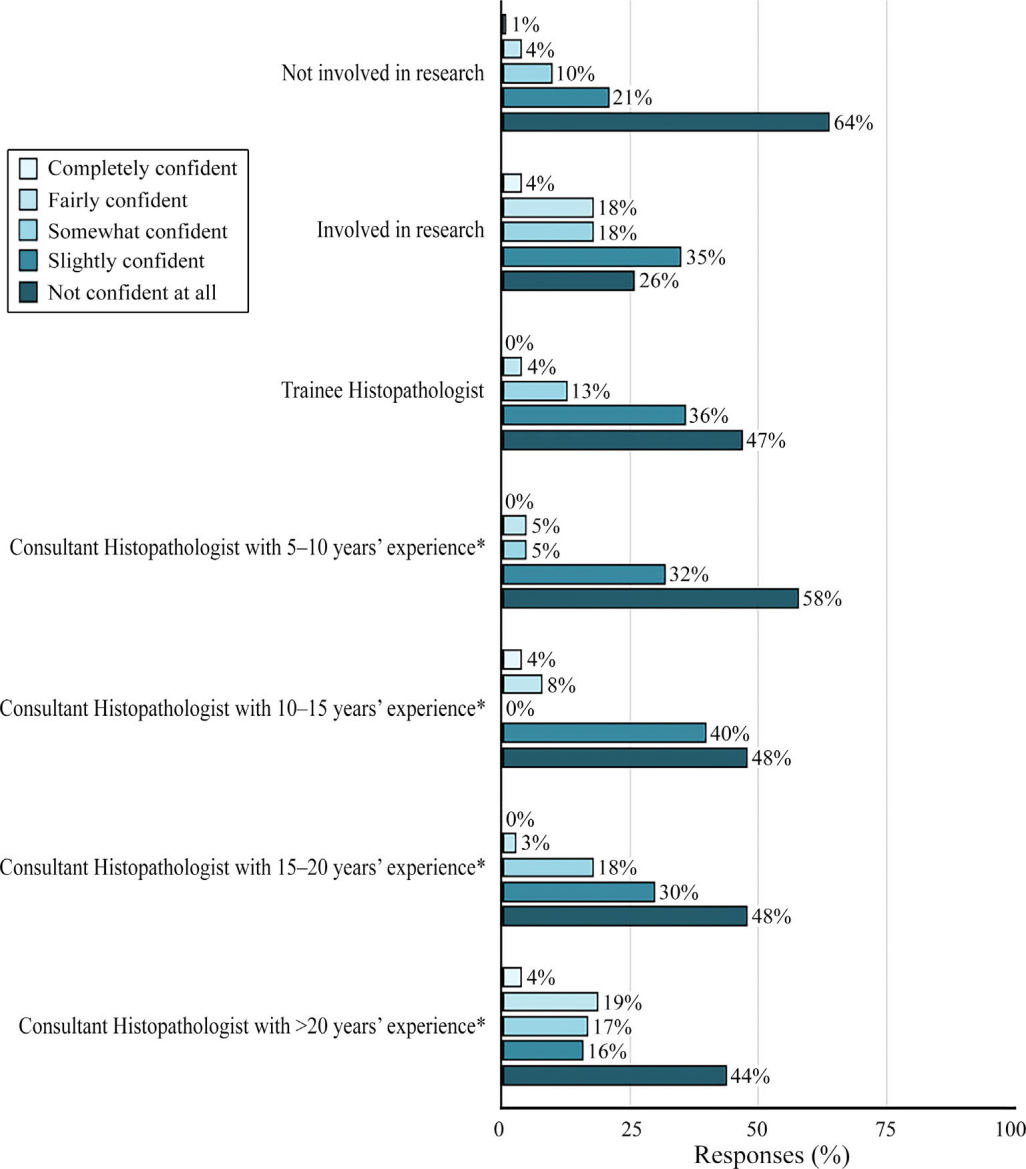
The percentage response rates to Question 15: ‘How confident do you feel in your understanding and interpretation of the appropriate use of consent in connection with scanned slide images in research’. *Or specialty doctor, including training.

When asked how confident respondents were in their understanding and interpretation of the appropriate use of consent in connection with scanned slide images in research, 47% (93/198) were ‘Not confident at all’ and only 2% (4/198) were ‘Completely confident’. Fewer research active respondents reported feeling ‘Not at all confident’ (26%, 22/85) compared with non‐research active respondents (64%, 65/101). These findings are summarised in Figure 2.

### Data sharing

Six percent (11/198) of all respondents had shared images outside of the EU and 7% (14/197) with industry, both for the purpose of research. On the issue of public awareness of data sharing with industry, 68% (133/195) of respondents thought that the public were ‘Not at all aware’ that anonymous data could be shared with industry. Two percent (3/195) thought they were ‘Completely aware’.

### Digitalisation of pathology

At the end of the e‐survey, we provided respondents with the opportunity to comment on the digitalisation of pathology. Analysis of their comments revealed four main themes: (1) governing data, (2) validating data, (3) Ownership and third‐party access, and (iv) inclusivity and transparency, with comments about governing data generating nearly 10 times more than anything else.

#### Governing data

Respondents commented on the need to maintain patient confidentiality and/or anonymity, if and when consent was needed and the need for data security (most notably, whether images were protected against hacking, breaches, or data loss during storage, transfer, or while working from home). Finally, this theme contained comments about the lack of, and need for, training on DP information governance.

#### Validating data

Respondents noted concerns around the risks and benefits of DP or AI‐driven diagnosis compared to traditional histopathology. Salient risks identified were the possibility for errors and misdiagnoses, the mixing up of slides, reproducibility of diagnosis, cost effectiveness of the new digital system, and the effects of digitalisation on pathologists' labour or behaviour. Regarding the latter, job loss, outsourcing, new liabilities, and overconfidence in AI were noted as possible negative outcomes of implementing digital workflows.

#### Ownership and third‐party access

Respondents raised questions about who owns data in the context of third‐party access, along with comments about the ethical risks of commercialisation and what constitutes a fair exchange for the sharing of DP data with for‐profit organisations.

#### Inclusivity and transparency

Respondents commented on the need for transparency about data uses and the inclusion of the views and opinions of the public in decisions about these uses. They suggested patients should be aware of how their data would be used and that patient preferences should be taken into consideration.

## Discussion

DP has genuine potential to develop an integrated global pathology community. The COVID‐19 pandemic has catalysed the urgency of DP adoption with the Food and Drug administration (FDA) relaxing restrictions to facilitate remote working for histopathologists and guidance on remote DP reporting being issued by the RCPath [57, 58]. The growth of interest in DP services highlights the urgent action needed to ensure that ethical/legal considerations do not lag behind the technical development.

Our survey has demonstrated that, despite growing DP use, histopathologists acknowledge a lack of confidence in their knowledge and understanding of the key issues of consent, legislation, and ethical guidelines. Although some of the issues raised are not in the domain for the practicing histopathologist, many are. It is important those working directly with the images are mindful that different governance considerations are required depending on the intended use. According to respondents' experiences, there is a lack of resources/training available to support histopathologists in navigating these areas (Table 3).

There is a paucity of specific guidance on the legal and ethical issues in the context of diagnostic DP. RCPath has professional guidance which ‘aims to give pragmatic and specific guidance on validation and verification of DP for clinical use’ and the focus is to assist the introduction of DP for primary diagnosis while maintaining safety [22]. Therefore, its purpose is not for research guidance or future applications and ethics falls outside of its remit. It includes a brief general legal section, highlighting the complex nature of such issues and advises involvement of information governance officers. It is important that support resources are highlighted, although an awareness of this area helps people to understand when it is appropriate to seek further help.

Globally, there are numerous country‐specific guidelines for the implementation and use of DP, at times more framed in the context of telepathology (Table 1). The issues of data governance and cybersecurity are raised, although often only discussed superficially in the context of implementation rather than specific day‐to‐day guidance and not always extended to alternative uses for the data beyond diagnostic reporting.

With regard to ethical guidance for diagnostic DP, the RCPath currently has no specific guidelines. However, focusing on the use of DP for AI development, there is a current drive by the UK Government to ‘create an ecosystem that ensures we get the use of AI “right” in health care’ through their development of NHSX [6]. A 2019 report explored a novel governance framework emphasising the softer ethical considerations of ‘should versus should not’ in the development of AI solutions as well as legislative regulations of ‘could versus could not’. It integrates the 2018 *Code of Conduct for Data‐Driven Health and Care Technology* from the Department of Health and Social Care, which aims to promote the development of AI with the Nuffield Council on Bioethics' *Principles for Data Initiatives* [6, 19, 59]. Although the above guidance exists, it is not specific to DP but AI, however the majority of our respondents were unaware of it.

More specific ethical guidance is available in Canada, which has a clearly established WSI workflow integrated with telepathology and a specific ‘*Code of ethics for storage and transmission of electronic laboratory data*’ [40]. Additionally, in 2005, updated in 2013, the Canadian Standards Association (CSA) developed a voluntary code based on the work of the ‘*Guidelines Governing the Protection of Privacy and Transborder Flows of Personal Data’*, created by the international ‘Organization for Economic Cooperation and Development (OECD)’. This has been endorsed by many Canadian companies as the national standard on privacy protection [39].

Understanding and implementing guidelines depend on background knowledge of the area. The acquisition of even a background level of understanding of these topics appears to be limited. From the authors' experience, available courses include generic governance/GDPR, with content not directly applicable to pathology.

The GDPR is legislation in force in the EU for the processing of personal data, either within the EU or such data related to people in the EU [28] (supplementary material, Appendix S1 – Question 9). The DPA 2018 is the UK's implementation of the GDPR (supplementary material, Appendix S1 – Question 9) [23]. Under these regulations, personal data (‘information related to an identified or identifiable living individual’) is classed as special category personal data when it includes health data [23, 28]. The application of the GDPR in the context of DP research is dependent on the data being processed, e.g. fully anonymised data does *not* fall under the ambit of the GDPR, whereas pseudonymised data may [28].

In terms of our questionnaire (supplementary material, Appendix S1 – Question 13), the use of digital scanned slides images falls under the relevant UK data protection legislation if there is (1) a patient name and *potentially* (2) a histology (accession) number on the slide, or (3) the slide is pseudonymised. We say ‘potentially’ as the ICO is currently undertaking a review of its anonymisation guidance and a first draft has been shared for consultation [60]. It states ‘Data protection law does not explicitly define “anonymous information”’ [60]. It goes on to state that ‘In the ICO's view, the same information can be personal data to one organisation, but anonymous information in the hands of another organisation. Its status depends greatly on its circumstance’ [60]. Once finalised and published, this new guidance may assist researchers to navigate this complex area. Until the guidance is published, a cautious approach is advised, and local information governance teams should be consulted.

According to the NHSX document *AI – How to get it right*, the ‘data access stage’ can also cause a lot of confusion, especially in determining the legal basis for processing data (direct patient care or secondary) and if their project should be classed as research or not. For instance, developing a piece of software using medical data should always be considered as a secondary use regardless of whether that software is eventually used to provide direct care to the patient. It should also be classed as research and having approval obtained from the Health Research Authority (HRA) [6]. The legal basis for data processing under the GDPR for AI‐based pathology research when using anonymised images is not consent as in many other patient settings but often legitimate interest under Article 6 of the GDPR. Under certain circumstances, if it is judged that the GDPR/DPA applies then, in the UK, a data protection impact assessment may need to be completed [28].

In the UK, the *Common Law Duty of Confidentiality* guidelines apply to personal information and are separate from data protection legislation (supplementary material, Appendix S1 – Question 9). They state that information given in confidence must not be shared with a third party without an individual's valid consent (or some other legal basis) [26]. This includes data that are anonymised as such data would be derived from a data set that would require consent from a data subject. There is also the application of the *National Data Opt‐Out*, which is a service that ‘allows patients to opt out of their confidential patient information being used for research and planning’, although this does not apply to anonymised information [61] (supplementary material, Appendix S1 – Question 9).

Difficulties interpreting legislation and guidelines can be compounded for researchers conducting multi‐centre, multi‐jurisdictional studies [62]. There is a drive to develop International Best Practice Guidance with involvement from the Global Digital Health Partnership (GDHP) – a collaboration of world governments and the WHO [63]. The WHO has also partnered with the ITU to establish an FG‐AI4H with the aim of identifying opportunities for international standardisation [6, 64].

Along with the appropriate application of legislation and guidelines, understanding when REC approval is needed is also essential for DP research. REC approval is required for any image analysis projects using patient data and therefore would NOT usually be required for diagnostic reporting, teaching or training, audit, service improvement, quality control or quality assurance, or use in an EQA (supplementary material, Appendix S1 – Question 14). The UK 2019 *State of the Nation Survey* built up a picture of critical issues surrounding ethics and regulation [6]. It revealed a 50/50 split in whether AI developers sought ethical approval. The current complex governance framework and lack of clarity around the development of AI technologies may be impeding innovation. Indeed, one of the NHSX's objectives is to ensure that in all future funding applications the expectation of ethical compliance is made clear. We acknowledge that some of the issues in our survey are more relevant to pathologists undertaking research. However, it is useful for all pathologists to be aware of them so that data are not inadvertently used for secondary purposes without the appropriate permissions.

A final consideration for DP research is the issue of data sharing. The majority of respondents in our e‐survey felt that the public were unaware that their anonymised data could potentially be shared with third parties. A 2020 UK survey of 2095 individuals found that 63% were unaware that the NHS gives third parties access to data [65]. Research has shown that the sharing of data can be viewed positively by patients, subject to the expectation that such data will be used to further the common good, is transparent, and benefits should be shared [65]. NHS, academia, and industry partnerships can bring synergistic skills into a collaboration, as industry often has greater experience of gaining regulatory approvals, potentially accelerating development to patient benefit [66]. The NHSX recognises people are neither aware that information within their health records has enormous research potential nor how it can be used in practice [54]. There are significant public concerns that commercial organisations may harvest and use their data [6]. Beyond the scope of this paper, there is also the issue of the NHS being given perpetual fair value for the use of data.

The focus of the survey was DP rather than AI, although these fields are inherently linked because WSI creates the raw data for building algorithms. Some respondents raised concerns about AI‐driven diagnosis, such as overconfidence in its performance. Algorithm development faces ethical challenges such as avoiding bias, maintaining openness about which data sets are used, and making it as clear as possible to users how an algorithm works. These challenges again underline the need for training histopathologists to understand and explain results from AI tools. It would be prudent to run this process in parallel with the DP roll out.

It is beyond the scope of this article to provide guidance on specific legal or ethical questions, and we intend only to present the general background, considerations, and potential issues that may arise in the DP setting. We also acknowledge that there may be no clear answer to some questions raised.

We have emphasised that the governance, legal, and ethical frameworks underpinning DP are complex and there is a lack of confidence in these areas. Highlighting these issues should not hinder the development of DP but instead help to build solid foundations for a safe and secure platform for our patients. Histopathologists should be specifically trained and assisted to help them navigate these areas. Specific training resources calling attention to the complexities should be developed to support histopathologists that are utilising DP. Finally, there were issues raised in this study, such as data commercialisation, cybersecurity, and the role of industry, which are outside the scope of this article but that clearly warrant further study in their own right.

## Author contributions statement

CC, LB and CV devised the initial concept. CC, CV, LB, RC and PM designed and developed the initial pilot e‐survey and subsequent analysis. CC, CV, FM, NH, LB, RC, PM, MA, DT, TS and GB were all involved in the design and development of the e‐survey. CC constructed the survey using the online platform and, along with CV, tested the e‐survey prior to circulation. LB, CV and DT arranged circulation of the e‐survey. CC, NH and FH were involved in the collection and collation of results. All authors were involved in the analysis of results, writing and editing the paper, and reviewed the article prior to submission.

## Supporting information


**Appendix S1.** Questionnaire
