# Supplementary material for: Understanding the ethical and legal considerations of Digital Pathology
Source: J Pathol Clin Res. 2021 Nov 18;8(2):101–15. doi: 10.1002/cjp2.251 (PMC8822384; doi:10.1002/cjp2.251)
Supplement: Supplementary file 1 — Appendix S1. Questionnaire [file CJP2-8-101-s001.pdf]

**Understanding the ethical and legal considerations of Digital Pathology**

C Coulter *et al.* *J Pathol Clin Res* DOI: 10.1002/cjp2.251

**Appendix S1.** Questionnaire

## Digital Pathology – Legal and ethical considerations

Digital Pathology is the acquisition, management, sharing and interpretation of pathology information including slides and data, in a digital environment [1]. This advancement enables the rapid transfer of data on potentially a global scale with the aim of facilitating and expediting research, developing Artificial Intelligence (AI) platforms and aiding clinical decision making. However, with the increased access to patients data in the digital environment there are many legal and ethical factors to consider.

We wish to ascertain the current level of understanding and confidence of UK histopathologists in the legal and ethical aspects of Digital Pathology, in order to establish future training needs and assist the development of the field.

We would be grateful if you could complete the following questionnaire. There are 20 questions in total, and we estimate it should take about 5-10 minutes to complete. All responses are fully anonymised and cannot be traced back to individuals or departments. Once the results are analysed, we aim to publish the survey results in a peer reviewed journal.

Thank you.

---

Reference [1] https://www.rcpath.org/discover-pathology/public-affairs/digital-pathology.html.

1) How would you best describe your current level of experience as a histopathologist?

(Please tick one of the following)

[ ] I am a consultant histopathologist or specialty doctor with >20 years' experience (including training)

[ ] I am a consultant histopathologist or specialty doctor with 15-20 years' experience (including training)

[ ] I am a consultant pathologist or specialty doctor with 10-15 years' experience (including training)

[ ] I am a consultant pathologist or specialty doctor with 5-10 year' experience (including training)

[ ] I am a trainee histopathologist

[ ] Other (please specify)

2) Where are you currently based?

[ ] England

[ ] Scotland

[ ] Wales

[ ] Northern Ireland

3) Which best describes your post? (Please tick the most relevant)

[ ] NHS post, no funded academic time

[ ] NHS post, including some funded academic time

[ ] Academic post including funded NHS time

[ ] Academic post with no funded NHS time

[ ] Other (please specify)

4) How would you best describe your current centre? (Please tick the most relevant)

[ ] NHS - district general hospital

[ ] NHS – tertiary referral centre

[ ] NHS centre and university academic department

[ ] University academic department

[ ] Private laboratory

[ ] Other (please specify)

5) What personal experience do you have of Digital Pathology (Please tick all that apply)

[ ] Primary diagnosis

[ ] Second opinion

[ ] Multi-disciplinary team (MDT) meeting

[ ] Research or clinical trials

[ ] Teaching or training

[ ] External Quality Assurance (EQA)

[ ] None of the above

[ ] Other (please specify)

6) Does your NHS trust offer mandatory training in information governance?

[ ] Yes

[ ] No

[ ] Not sure

7) Have you undertaken additional training in information governance? (I.e. online modules, information /- data governance conferences)

[ ] Yes

[ ] No

If yes, please comment on format, length, content and relevance?

8) Would you like to receive additional training in information governance?

[ ] Yes

[ ] No

If yes, what delivery and content would you like?

9) How confident do you feel regarding your knowledge and understanding of the following policies and/ or legislation in the setting of digital pathology?

**General Data Protection Regulation (GDPR)**

[ ] Not confident at all [ ] Slightly confident [ ] Somewhat confident [ ] Fairly confident [ ] Completely confident

**Data Protection Act 2018**

[ ] Not confident at all [ ] Slightly confident [ ] Somewhat confident [ ] Fairly confident [ ] Completely confident

**Health and Social Act 2018**

[ ] Not confident at all [ ] Slightly confident [ ] Somewhat confident [ ] Fairly confident [ ] Completely confident

**EU-US Privacy Shield**

[ ] Not confident at all [ ] Slightly confident [ ] Somewhat confident [ ] Fairly confident [ ] Completely confident

**NHS National Data Opt out**

[ ] Not confident at all [ ] Slightly confident [ ] Somewhat confident [ ] Fairly confident [ ] Completely confident

**Common Law Duty of Confidentiality**

[ ] Not confident at all [ ] Slightly confident [ ] Somewhat confident [ ] Fairly confident [ ] Completely confident

[ ] I am not aware of any of these

[ ] Other not listed above [please specify]

<u>10) How confident do you feel regarding your knowledge and understanding of the following pieces of ethical guidance?</u>

**Code of Conduct for Data Driven Health and Care Technology 2018**

[ ] Not confident at all [ ] Slightly confident [ ] Somewhat confident [ ] Fairly confident [ ] Completely confident

**NHS X: Artificial Intelligence: How to Get it Right. 2019**

[ ] Not confident at all [ ] Slightly confident [ ] Somewhat confident [ ] Fairly confident [ ] Completely confident

**Data Ethics Framework by Department for Digital Culture, Media and Sport**

[ ] Not confident at all [ ] Slightly confident [ ] Somewhat confident [ ] Fairly confident [ ] Completely confident

[ ] I am not aware of any of these

[ ] Other not listed above (please specify)

<u>11) Are you aware of any ethical and/or legal considerations for Digital Pathology that are unique to your region (England/Scotland/Wales/Northern Ireland) when compared to the rest of the UK?</u>

[ ] Yes

[ ] No

If yes, please provide details.

<u>12) Have you been involved in any research using digital scanned slides?</u>

[ ] Yes

[ ] No

[ ] No, but I plan to

<u>13) In research, when would the use of digital scanned slide images fall under the relevant UK data protection legislation (GDPR & Data Protection Act 2018)? (Please tick all that apply)</u>

[ ] It always does

[ ] If there is a patient name on the slide label

[ ] If there is a histology (accession) number on the slide

[ ] The slide is fully anonymised (link to the case permanently broken)

[ ] The slide is pseudo anonymised (personal identifiers are removed, but a link or key to identify the case remains)

[ ] I do not know

[ ] Other (please specify)

14) Which of the following activities, using digital scanned slides/ platform; would you **NOT** require Research Ethics Committee (REC) approval for? [Please tick all that apply]

[ ] For diagnostic reporting

[ ] For teaching or training

[ ] For audit

[ ] For service improvement

[ ] For quality control or quality assurance

[ ] For use in an External Quality Assurance (EQA)

[ ] For development of image analysis (including AI/machine learning/deep learning) if there is any personal data on the slide images

[ ] For development of image analysis (including AI/machine learning/deep learning) if the slide images are fully anonymised (link to the case permanently broken)

[ ] For development of image analysis (including AI/machine learning/deep learning) if the slide images are pseudo anonymised (a link or key to identify the case remains)

[ ] For any activity involving scanned slide images

[ ] No Research Ethics Committee (REC) approval needed for any of these activities

Please add any comments

15) How confident do you feel in your understanding and interpretation of the appropriate use of consent in connection with scanned slide images in research?

[ ] Not confident at all [ ] Slightly confident [ ] Somewhat confident [ ] Fairly confident [ ] Completely confident

Please add any comments

16) In your institution, is there a statement on the NHS consent to procedure form about use of data and/or tissue in research, to which patients can consent or specify opt-out? (please tick one)

[ ] Yes there is a statement about tissue research

[ ] Yes there is a statement about data research

[ ] Yes there is a statement for each of data and tissue research (separate consent statements/ options)

[ ] Yes there is a combined statement for data and tissue research (together as one consent statement/option)

[ ] There is no statement about tissue or data research

[ ] I don't know

[ ] Other [please specify]

17) In the context of research, have you shared any digital scanned slides/ images outside of the European Union?

[ ] Yes

[ ] No

[ ] Not Applicable – I have not conducted any research on digital scanned slides

If yes, please specify (i.e. context, who with, any agreements used)

18) In the context of research, have you shared any digital scanned slides/ images with third parties in industry outside of the NHS?

[ ] Yes

[ ] No

[ ] Not Applicable – I have not conducted any research on digital scanned slides.

If yes, please specify (i.e. context, who with, any agreements used)

19) How aware do you think the public are that their anonymous data may be shared with industry in order to facilitate developments in Digital Pathology e.g. to develop AI algorithms. [ ] Not at all [ ] A little aware [ ] Somewhat aware [ ] Completely aware

Please add any comments you may have about sharing scanned slide images with industry

20) Please list from most important to least important; what you consider as up to three key legal and/or ethical issues surrounding Digital Pathology, for diagnostics and/or research, in terms of data/information.

[1.]

[2.]

[3.]

21) Please list from most important to least important what you consider are three benefits that might be gained from widespread Digital Pathology use?

[1.]

[2.]

[3.]

22) Please add any further comments, issues or concerns that have not been discussed.

---

**Thank you for taking the time to complete this questionnaire.**
